# An Efficient Closed Form Solution to the Absolute Orientation Problem for Camera with Unknown Focal Length

**DOI:** 10.3390/s21196480

**Published:** 2021-09-28

**Authors:** Kai Guo, Hu Ye, Zinian Zhao, Junhao Gu

**Affiliations:** Northwest Institute of Nuclear Technology, Xi’an 710024, China; yxc0228@163.com (H.Y.); zhao_9711@163.com (Z.Z.); gujunhao13@163.com (J.G.)

**Keywords:** absolute orientation, camera position, angle constraint, single solution, unknown focal length, perspective-three-point

## Abstract

In this paper we propose an efficient closed form solution to the absolute orientation problem for cameras with an unknown focal length, from two 2D–3D point correspondences and the camera position. The problem can be decomposed into two simple sub-problems and can be solved with angle constraints. A polynomial equation of one variable is solved to determine the focal length, and then a geometric approach is used to determine the absolute orientation. The geometric derivations are easy to understand and significantly improve performance. Rewriting the camera model with the known camera position leads to a simpler and more efficient closed form solution, and this gives a single solution, without the multi-solution phenomena of perspective-three-point (P3P) solvers. Experimental results demonstrated that our proposed method has a better performance in terms of numerical stability, noise sensitivity, and computational speed, with synthetic data and real images.

## 1. Introduction

Many methods have been proposed to estimate absolute camera pose, i.e., the position and orientation, such as the perspective-n-point (PnP) solver [1,2,3,4,5,6,7,8,9], which uses *n* known 2D–3D point correspondences. Pose estimation is one of the key steps in computer vision [2,10,11], photogrammetry [3,11,12], augmented reality (AR) [4,13,14,15], structure from motion (SfM) [4,14,16], multi-view 3D reconstruction [17,18], and simultaneous localization and mapping (SLAM) [4,8,13,19]. The absolute pose of a fully uncalibrated camera pose contains six unknown parameters, and each 2D–3D point correspondence gives two constraints [20], which means that the P3P is the minimal subset to determine the camera pose if the position and orientation are both unknown [10,21,22,23,24]. Many P3P solvers have been proposed, and all the solvers have up to four possible solutions [12,25,26]. In general, disambiguating the multi-solution phenomena can be done by using a fourth point. We can see that, although the P3P needs minimal 2D–3D point correspondences, all P3P solvers have some disadvantages: a fully calibrated camera is needed and multi-solution phenomena exists. These disadvantages thus prevent their application when the intrinsic camera parameters change online or are unknown. Hence, for pose estimation, many methods have been proposed to work with a partially calibrated camera and more 2D–3D point correspondences [27]. Some methods, namely the PnPf solvers, work well with cases of unknown focal length [28,29,30]. Four or more 2D–3D point correspondences are needs for all PnPf solvers. The P4Pf is the minimal subset, and different methods have been proposed to focus on the planar case [31], the non-planar case [27], or both [32]. Compared to the P3P solvers, only one more parameter, i.e., focal length, must be obtained, and they are iterative algorithms or need to solve quadratic or quadric polynomial equations of several variables. Hence, some methods have been proposed to work with unknown focal length and unknown radial distortion (namely, the PnPfr solvers [33,34]), while some work with unknown focal length and unknown aspect ratio [35], or unknown focal length and unknown principal point [27]. When *n* ≥ 6, the pose estimation can be linearly estimated, known as direct linear transform (DLT) [18,32], and all the parameters of a fully uncalibrated camera can be obtained.

Note that more parameters can be estimated with more 3D control points. However, in some cases, not enough 3D control points can be obtained because accurate 3D control points are expensive to acquire and maintain. This requires us to use as few points as possible to estimate the pose with a partially calibrated camera, and there are two ways to reduce the number of the 3D control points in existing PnP solvers. The first way is to use some prior knowledge of the intrinsic camera parameters. For most modern digital cameras, the aspect ratio of the pixels, the skew, and the principal point are known and do not change [32,33]; hence, these parameters can be assumed as prior knowledge, which means we can use fewer 3D control points to estimate the remaining unknown parameters. With this assumption, only the focal length is unknown of the intrinsic camera parameters, and it will be shown that, in our experiments and practical application, this assumption works well, even though it is not always strictly met.

In addition, since modern digital cameras can be equipped with various positioning and orientation sensors, the second method is to measure some pose parameters in advance, as prior knowledge. Some methods focus on the pose problem with the known vertical direction. This can be obtained directly using orientation sensors, such as gyroscopes, accelerometers, or inertial measurement units (IMUs) [3,20,36,37,38,39,40,41]. The vertical direction can give knowledge of the orientation of roll and pitch, which means only four pose parameters are left to be estimated [13,15,17,42,43,44,45]. These methods can use two 3D points for pose problem and give two solutions. Some methods solve the pose problem with three 2D–3D point correspondences and the vertical direction. In this case, six parameters (one orientation parameter, three position parameters, radial distortion, and focal length) can be determined with a single solution.

In this paper, the idea is also to measure some pose parameters in advance, as prior knowledge, but not the orientation parameters. Pose parameters include the orientation and position. However, to the best of our knowledge, almost all recent research has focused on the known orientation parameters, and very few works focused on the known position parameters. Moreover, in some cases, the camera position and 3D control point positions can be obtained accurately as prior knowledge using a positioning device (e.g., RTK, total station). In a missile testing range, for example, altitude measurement based on fixed cameras is an important test. These cameras are fixed and for absolute pose problem, some 3D control points in the world frame must be exactly known. Hence, in this paper, we focus on the known position parameters [46] to solve the pose problem, and we give an efficient closed form solution to the absolute orientation problem with unknown focal length from two 2D–3D point correspondences. Since each point correspondence can give us two constraints [3], this is the minimum number of point correspondences needed to estimate the absolute orientation and focal length in this case. Here, the problem can be decomposed into two sub-problems and can be solved with angle constraints. Rewriting the camera model with the known camera position leads to a simpler and more efficient method for pose estimation, and it gives a single solution, without the multi-solution phenomena of existing P3P solvers.

The rest of this paper is organized as follows. In Section 2, we propose our method to efficiently estimate the focal length and the absolute orientation. In Section 3, we present a thorough analysis of our proposed method with synthetic data and real images, compared to some other existing PnP solvers. In Section 4, we present the discussion. In Section 5, we present the conclusions. 

## 2. Materials and Methods

**Figure 1:**
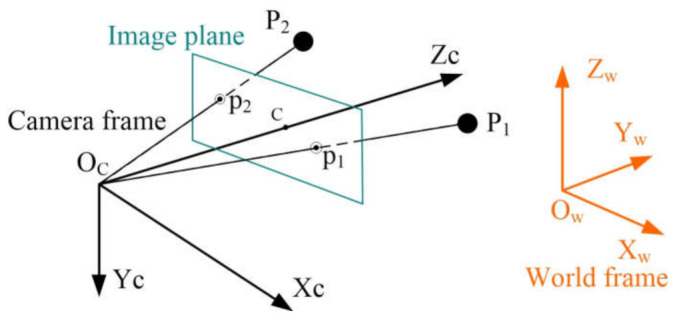
Standard pinhole camera model with two 3D control points. Here C is the principal point.

In this paper, we propose an efficient closed form solution to the absolute orientation problem for cameras with unknown focal length from two 2D–3D point correspondences and the camera position. The standard pinhole camera model [18] is used, as shown in Figure 1. In our problem, we assume that the skew is zero, the aspect ratio of the pixels is one, and the principal point is the center of the image, which is true for most modern digital cameras and can yield good results, even when they are not exactly satisfied; as will be shown in the experiments [3,33]. In this paper, the camera position $O_{c}\left(X_{Oc},Y_{Oc},Z_{Oc}\right)$ is known, which can be obtained by positioning sensors [45,47] or measured by the total station [48].

In Figure 1, 3D points $P_{i}\left(X_{wi},Y_{wi},Z_{wi}\right),\;i=1,2$ in the world frame *O_ZYZ_w* are projected onto 2D image points $p_{i}\left(u_{i},v_{i}\right)$ on the camera image plane. This can be written as

(1) $$\lambda_{i}\left[\begin{array}{c} u_{i}\\ v_{i}\\ 1 \end{array}\right]=M\left[\begin{array}{c} X_{wi}\\ Y_{wi}\\ Z_{wi}\\ 1 \end{array}\right]$$

In this equation, *M* is a 3 × 4 camera projection matrix and $\lambda_{i}$ is an unknown scale factor. From the standard pinhole camera model, *M* can be written as

(2) $$M=K\left[R|t\right]$$

Here, *K* is a 3 × 3 camera calibration matrix that contains the focal length information. *R* and *t*, which contain all the pose information, are respectively a 3 × 3 rotation matrix and a 3 × 1 translation vector from the world frame to the camera frame. Our problem is to estimate *R*, *t,* and the focal length *f* from two 2D–3D point correspondences. Next, we propose our method to estimate the focal length and absolute orientation with angle constraints.

### 2.1. Closed Form Solution to the Focal Length

In this paper, we assume

(3) $$K=\left[\begin{array}{ccc} f & 0 & 0\\ 0 & f & 0\\ 0 & 0 & 1 \end{array}\right]$$

Then we can obtain the vector $\vec{O_{c}p_{i}}$ in the camera frame

(4) $$\vec{O_{c}p_{i}}=\left[u_{i},v_{i},f\right]$$

**Figure 2:**
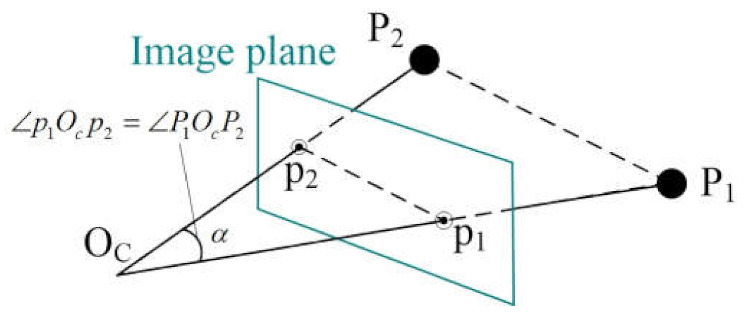
Angle constraint for the focal length estimation.

From Figure 1, the angle constraint now can be used to estimate the focal length, as illustrated in Figure 2. 

With the positions of 3D point $P_{1},P_{2}$ and the camera position $O_{c}$ in the world frame, we can obtain the vector $\vec{O_{c}P_{i}}$

(5) $$\vec{O_{c}P_{i}}=P_{i}-O_{c}$$

Then, $∠P_{1}O_{c}P_{2}$ can be computed as α

(6) $$\alpha =\arccos\frac{\vec{O_{c}P_{1}}\cdot \vec{O_{c}P_{2}}}{\left\|\vec{O_{c}P_{1}}\right\|\cdot \left\|\vec{O_{c}P_{2}}\right\|}$$

In the camera frame, from Equation (4) $∠p_{1}O_{c}p_{2}$ can be computed and from Figure 2, we can see $∠p_{1}O_{c}p_{2}=∠P_{1}O_{c}P_{2}$, which leads to the equation

(7) $$\cos\alpha =\frac{\vec{O_{c}p_{1}}\cdot \vec{O_{c}p_{2}}}{\left\|\vec{O_{c}p_{1}}\right\|\cdot \left\|\vec{O_{c}p_{2}}\right\|}=\frac{u_{1}u_{2}+v_{1}v_{2}+f^{2}}{\sqrt{u_{1}^{2}+v_{1}^{2}+f^{2}}\cdot \sqrt{u_{2}^{2}+v_{2}^{2}+f^{2}}}$$

We let $f^{2}=a$, $u_{1}u_{2}+v_{1}v_{2}=b$, $u_{1}^{2}+v_{1}^{2}=c$ and $u_{2}^{2}+v_{2}^{2}=d$. From Equation (7), a quadratic equation with one variable, i.e., a, can be given

(8) $$\left(1-\cos^{2}\alpha \right)a^{2}+\left(2b-c\cdot \cos^{2}\alpha -d\cdot \cos^{2}\alpha \right)a+b^{2}-cd\cdot \cos^{2}\alpha =0$$

Two possible solutions to a can be obtained. Then up to four possible solutions to the focal length can be given from Equation (8). Note that a>0, f>0, and cosα>0, then a single closed form solution can be given.

### 2.2. Pose Estimation with Angle Constraint

**Figure 3:**
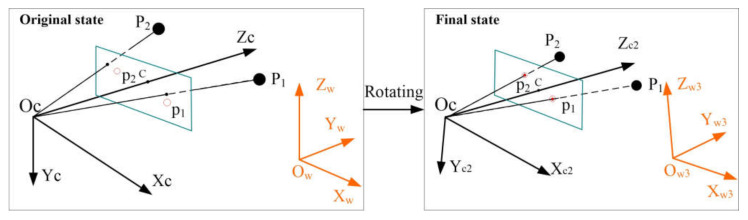
Rotating for pose estimation.

In this paper, we first place the camera with an original known pose in the world frame, which means the transformation between the camera frame and the world frame is known. Then the pose estimation is obtained through rotating the camera and world frame to make the camera position *Oc*, 2D image point $p_{i}$ and 3D point *P_i_* collinear. The process is illustrated in Figure 3.

In the original state, the camera pose is known in the original world frame *O_ZYZ_w*; however, the 2D image point $p_{i}$ and 3D point *P_i_* have no correspondence, as shown in Figure 3 (left). The main work is to rotate the original camera frame *O_ZYZ_c* and world frame *O_ZYZ_w* to make the camera position *Oc*, 2D image point $p_{i}$, and 3D point *P_i_* collinear in the final state, as shown in Figure 3 (right).

Now we formulate the absolute orientation estimation problem as follows:

(1) Finish the 2D–3D point correspondence between point $p_{1}$ and point *P*_1_. In the original camera frame *O_ZYZ_c*, the *Xc*-axis and *Zc*-axis are parallel with the *X*-axis and *Y*-axis of the original world frame *O_ZYZ_w* in the same direction, and the *Yc*-axis is parallel with the *Z*-axis in the opposite direction. Then the position of point *P*_1_ in the camera frame *O_ZYZ_c*, which is named $P_{1}^{c}$, can be obtained using the formula

(9) $$P_{1}^{c}=R_{ox}\cdot \left[P_{1}-O_{c}\right]$$

Here,

(10) $$R_{ox}=\left[\begin{array}{ccc} 1 & 0 & 0\\ 0 & \cos90{}^{\circ} & -\sin90{}^{\circ}\\ 0 & \sin90{}^{\circ} & \cos90{}^{\circ} \end{array}\right]=\left[\begin{array}{ccc} 1 & 0 & 0\\ 0 & 0 & -1\\ 0 & 1 & 0 \end{array}\right]$$

The position of point $p_{1}$ in the camera frame *O_ZYZ_c*, meanwhile, which is named $p_{1}^{c}$, can be obtained using the formula

(11) $$p_{1}^{c}={\left[\begin{array}{ccc} u_{1} & v_{1} & f \end{array}\right]}^{T}$$

In the camera frame, we rotate the camera around the *Yc*-axis to make the projections of $\vec{O_{c}P_{1}^{c}}$ and $\vec{O_{c}p_{1}^{c}}$ onto the plane $Y_{c}=0$ collinear. The rotation angle $A_{Y_{c}}$ can be obtained using the formula

(12) $$A_{Y_{c}}=\arccos{\left(\frac{\vec{O_{c}P_{1}^{c}}\cdot \vec{O_{c}p_{1}^{c}}}{\left\|\vec{O_{c}P_{1}^{c}}\right\|\left\|\vec{O_{c}p_{1}^{c}}\right\|}\right)}_{Y_{c}=0}$$

After the first rotation, a new camera frame *O_ZYZ_c*1 is obtained and in this frame, the position of point *P*_1_, named $P_{1}^{c1}$, can be written as

(13) $$P_{1}^{c1}=R_{cY_{c}}\cdot P_{1}^{c}$$

Here,

(14) $$R_{cY_{c}}=\left[\begin{array}{ccc} \cos A_{Y_{c}} & 0 & -\sin A_{Y_{c}}\\ 0 & 1 & 0\\ \sin A_{Y_{c}} & 0 & \cos A_{Y_{c}} \end{array}\right]$$

The position of point $p_{1}$ in the new camera frame, named $p_{1}^{c1}$, is unchanged, which means $p_{1}^{c1}=p_{1}^{c}$. 

**Figure 4:**
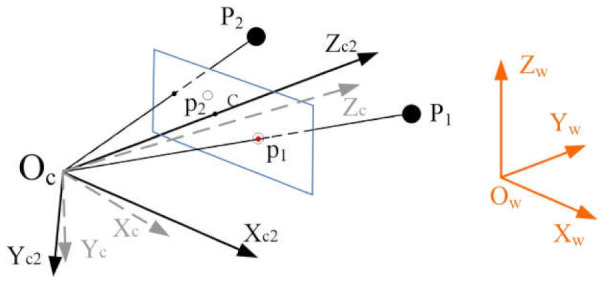
Camera frame *O_ZYZ_c*2. Now the 2D–3D point correspondence between point $p_{1}$ and point *P*_1_ is finished.

Next, we rotate the camera around the *Xc*_1_-axis to make $\vec{O_{c}P_{1}}$ and $\vec{O_{c}p_{1}}$ collinear. Now we obtain another camera frame *O_ZYZ_c*2, as shown in Figure 4.

The rotation angle $A_{X_{c1}}$ can be obtained using the formula

(15) $$A_{X_{c1}}=\arccos{\left(\frac{\vec{O_{c}P_{1}^{c1}}\cdot \vec{O_{c}p_{1}^{c1}}}{\left\|\vec{O_{c}P_{1}^{c1}}\right\|\left\|\vec{O_{c}p_{1}^{c1}}\right\|}\right)}_{X_{c1}=0}$$

The 2D–3D point correspondence between point $p_{1}$ and point *P*_1_ is completed as shown in Figure 4.

(2) Finish the 2D–3D point correspondence between point $p_{2}$ and point *P*_2_. When the point correspondence between point $p_{2}$ and point *P*_2_ is finished and the point correspondence between point $p_{1}$ and point *P*_1_ is unchanged, the camera absolute orientation is obtained. 

Now the position of point $p_{2}$ in the original world frame *O_ZYZ_w*, named $p_{2}^{w}$, can be computed with

(16) $$p_{2}^{w}={\left(R_{cX_{c1}}\cdot R_{cY_{c}}\cdot R_{ox}\right)}^{-1}\cdot \left[\begin{array}{c} u_{2}\\ v_{2}\\ f \end{array}\right]+O_{c}$$

In this equation,

(17) $$R_{cX_{c1}}=\left[\begin{array}{ccc} 1 & 0 & 0\\ 0 & \cos A_{X_{c1}} & -\sin A_{X_{c1}}\\ 0 & \sin A_{X_{c1}} & \cos A_{X_{c1}} \end{array}\right]$$

To maintain the point correspondence between point $p_{1}$ and point *P*_1_, we rotate the original world frame around the line *OcP*_1_. We thus define a new world frame, *O_ZYZ_w*1, whose origin *Ow*_1_ is camera position *Oc*, and

(18) $$\begin{array}{l} \vec{O_{w1}X_{w1}}=\frac{\vec{O_{w1}P_{1}}}{\left\|\vec{CP_{1}}\right\|}\\ \vec{O_{w1}Z_{w1}}=\frac{\vec{O_{w1}P_{1}}\times \vec{O_{w1}P_{2}}}{\left\|\vec{O_{w1}P_{1}}\times \vec{O_{w1}P_{2}}\right\|}\\ \vec{O_{w1}Y_{w1}}=\vec{O_{w1}Z_{w1}}\times \vec{O_{w1}X_{w1}} \end{array}$$

**Figure 5:**
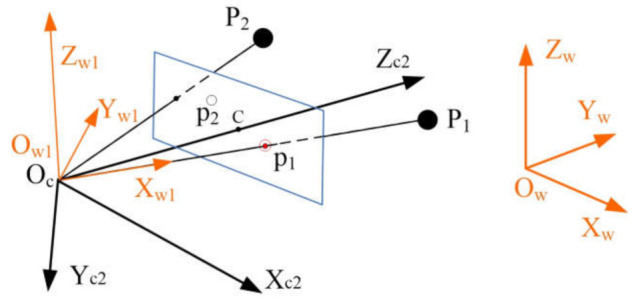
New world frame *O_ZYZ_w*1. The *Xw*_1_-axis is collinear with the line *OcP*_1_.

The new world frame *O_ZYZ_w*1 is illustrated in Figure 5.

In the new world frame *O_ZYZ_w*1 the positions of point *P*_2_ and $p_{2}$ can be given with

(19) $$\begin{array}{l} P_{2}^{w1}=R_{w1}\left(P_{2}-O_{c}\right)\\ p_{2}^{w1}=R_{w1}\left(p_{2}^{w}-O_{c}\right) \end{array}$$

Here,

(20) $$R_{w1}={\left[\begin{array}{ccc} \vec{O_{w1}X_{w1}} & \vec{O_{w1}Y_{w1}} & \vec{O_{w1}Z_{w1}} \end{array}\right]}^{T}$$

We rotate the world frame *O_ZYZ_w*1, point *P*_1_, and *P*_2_ around the *Xw*_1_-axis. With this rotation the relative pose between the world frame and point *P_i_* is unchanged, while the relative pose between the world frame and the camera frame is changed. 

To make $\vec{O_{c}P_{2}}$ and $\vec{O_{c}p_{2}}$ collinear, we rotate the world frame *O_ZYZ_w*1 around the *Xw*_1_-axis with an angle

(21) $$A_{xw1}=\arccos\left(\frac{\vec{O_{w1}P_{2}^{w1}}\cdot \vec{O_{w1}p_{2}^{w1}}}{\left\|\vec{O_{w1}P_{2}^{w1}}\right\|\left\|\vec{O_{w1}p_{2}^{w1}}\right\|}\right)$$

After this rotation, another world frame *O_ZYZ_w*2 is obtained and the rotation matrix between the world frame *O_ZYZ_w*1 and the world frame *O_ZYZ_w*2 is written as

(22) $$R_{w2}=\left[\begin{array}{ccc} 1 & 0 & 0\\ 0 & \cos A_{xw1} & -\sin A_{xw1}\\ 0 & \sin A_{xw1} & \cos A_{xw1} \end{array}\right]$$

**Figure 6:**
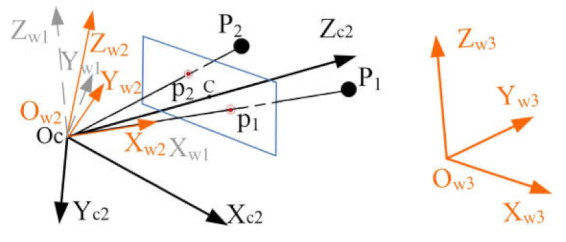
Two 2D–3D point correspondences in the final state. Now the absolute pose estimation is finished.

In addition, the original world frame *O_ZYZ_w* is changed to a new world frame *O_ZYZ_w*3. Finally, the two 2D–3D point correspondences are finished as shown in Figure 6.

**Figure 7:**
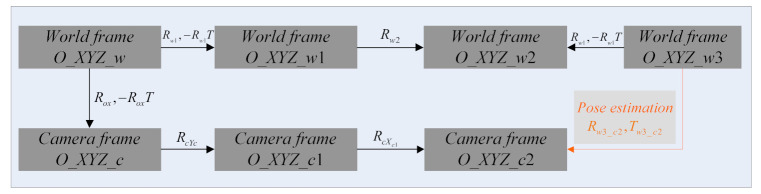
Transformations of all the frames. The transformation (yellow) between the world frame *O_ZYZ_w*3 and the camera frame *O_ZYZ_c*2 is unknown and needs to be estimated, while the other transformations have been computed.

(3) Estimate the absolute orientation. Several frames are involved in our proposed method, and now the transformations between each are known, except for the transformation between the world frame *O_ZYZ_w*3 and the camera frame *O_ZYZ_c*2, which is the very pose information that needs to be estimated in this paper. The transformations are shown in Figure 7.

Based on Figure 7, we can finally transform point $P_{i}^{w3}$ in the world frame *O_ZYZ_w*3 into point $P_{i}^{c2}$ in the camera frame *O_ZYZ_c*2 using

(23) $$\begin{array}{l} P_{i}^{c2}=R_{w3\_c2}\cdot P_{i}^{w3}+T_{w3\_c2}\\ R_{w3\_c2}=R_{cX_{c1}}\cdot R_{cY_{c}}\cdot R_{ox}\cdot R_{w1}^{-1}\cdot R_{w2}^{-1}\cdot R_{w1}\\ T_{w3\_c2}=-R_{w3\_c2}\cdot O_{c} \end{array}$$

The absolute orientation estimation with unknown focal length is finished.

## 3. Experiments and Results

We first tested the robustness to camera position noise of our proposed method with synthetic data.

Then we thoroughly tested our proposed method with synthetic data, including numerical stability, noise sensitivity, and computational speed, compared to other existing PnP solvers: the GP4Pf [28] and Kneip’s method [10]. The two existing PnP solvers both give up to four possible solutions, while we used one more point to give a single solution.

Lastly, our proposed method was tested with real images to show its performance in a practical application.

### 3.1. Synthetic Data

In this paper, the synthetic data consisted of three thousand 2D–3D point correspondences. Here, these 3D points were randomly distributed in a box of [−20, 20] × [−20, 20] × [180, 220] in the world frame. Then they were projected onto 2D points in the image plane using a virtual perspective camera, whose position was fixed at $O_{c}={\left[1,1,1\right]}^{T}$ and the angles in degree of the orientation were kept at $\left[roll,pitch,yaw\right]=\left[5,5,5\right]$. For the intrinsic parameters of the virtual perspective camera, the focal length was set to 50 mm and the image resolution was set to 1280 × 800 pixels.

For each trail, two 2D–3D point correspondences were randomly selected from the synthetic data for our proposed method, while three 2D–3D point correspondences were randomly selected from the synthetic data for Kneip’s method, and four 2D–3D point correspondences were randomly selected from the synthetic data for the GP4Pf. Moreover, one further 2D–3D point correspondence was selected for Kneip’s method and GP4Pf to disambiguate the multi-solution phenomena.

#### 3.1.1. Robustness to Camera Position Noise

Our proposed method uses the camera position as the prior knowledge, which is different from the existing methods. Therefore, the camera position is important, and it is necessary to analyze the effect of error in the camera position on the estimation of the absolute orientation and the focal length.

**Figure 8:**
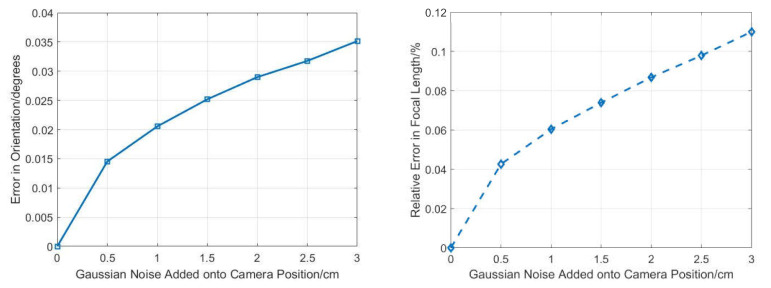
Robustness to camera position noise for orientation (**left**) and focal length (**right**).

The camera position is usually obtained by RTK or total station. In general, the measuring precision of RTK is better than 3 cm and the measuring precision of total station is better than 0.5 cm. Therefore, zero-mean Gaussian noise was added to the camera position and the noise deviation level varied from 0 to 3 cm. Next, 50,000 independent trails with two 2D–3D point correspondences of synthetic data were performed at each noise level. Then the average error of the absolute orientation and focal length were reported, as shown in Figure 8.

From Figure 8, we can see the orientation error and focal length error increase with the increase of camera position error. However, the max errors in orientation and focal length when the camera position error is 3 cm were both low, which means our proposed method has good robustness to camera position noise and still yields good results, even though camera position error existed.

#### 3.1.2. Numerical Stability

**Figure 9:**
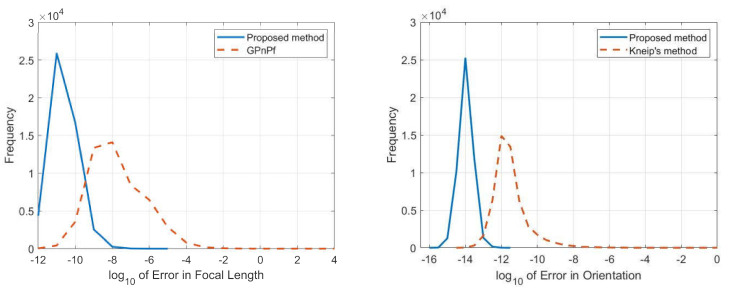
Relative error in focal length (**left**) and error in orientation (**right**) for our proposed method (blue) and the other methods (yellow).

In this section, 50,000 trails were performed independently and there was no noise added to the 2D–3D point correspondences. The log10 value of the relative error between the ground truth and the focal length, estimated using our proposed method and GP4Pf, respectively, is shown in Figure 9 (left). The log10 value of the error in orientation between the ground truth and the estimated value using our proposed method and Kneip’s method, respectively, is shown in Figure 9 (right).

From Figure 9 (left), the distribution of the log10 value of the relative focal length error can be observed. Clearly, our proposed method has much higher numerical stability than the GP4Pf. 

From Figure 9 (right), the distribution of log10 value of error in orientation can be observed. Obviously, our proposed method has much higher numerical stability than Kneip’s method.

#### 3.1.3. Noise Sensitivity

**Figure 10:**
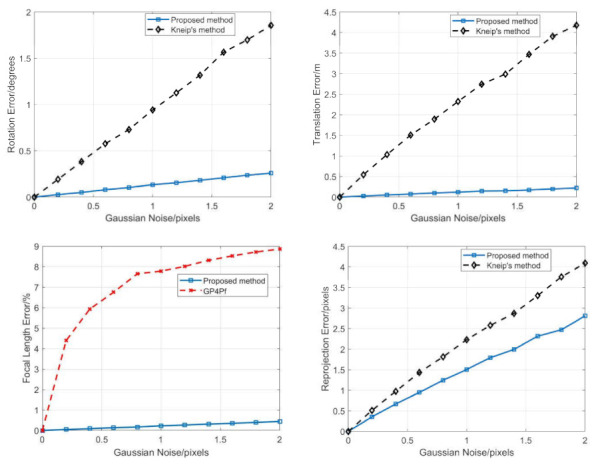
Average error of rotation (**top left**), translation (**top right**), focal length (**bottom left**) and reprojection (**bottom right**) for our proposed method (blue), Kneip’s method (black), and GP4Pf (red).

Zero-mean Gaussian noise was added to the 2D image points and the noise deviation level varied from 0 to 2 pixels. Then, 50,000 independent trails were performed at each noise level. The average error of the rotation, translation, focal length, and reprojection error were reported, as shown in Figure 10.

From Figure 10, in terms of the rotation and translation error, our proposed method performed much better than Kneip’s method, while it was slightly better in terms of reprojection error. In terms of the relative focal length error, our proposed method performed much better than the GP4Pf. Moreover, as the noise increases, the performance superiority of our proposed method over the other methods became more obvious.

#### 3.1.4. Computational Time

In this section, to analyze the computational time, 50,000 trails were executed independently on a 3.3 GHz 4-core laptop, and there was no noise added to the 2D–3D point correspondences. In each trial, note that one more point was needed to disambiguate multi-solution phenomena for Kneip’s method and the GP4Pf. The average computational time is reported in Table 1. 

We note that our proposed method performed much faster than the GP4Pf, while it was slightly faster than Kneip’s method.

### 3.2. Real Images

When we generated the synthetic data, the focal length and absolute orientation of the virtual perspective camera were ground truth. Therefore, we could make direct comparisons, leading to direct results. However, in the real-image experiments, we fixed a high-speed camera with a zoom lens on a tripod, and set the focal length to roughly 50 mm. This meant that the ground truth of the focal length and absolute orientation could not be directly and accurately measured by direct physical measurement. Although many methods have been proposed to estimate the focal length and absolute orientation, these are just measured values, not the ground truth.

Although the focal length and absolute orientation cannot be directly and accurately measured by direct physical measurement, the spatial position of the points can be directly and accurately measured by direct physical measurement (total station). The world frame can be established by total station in the lab, and the measurement accuracy of total station is generally better than 0.5 cm. Therefore, in this paper we took the spatial position of a point measured by total station as the ground truth, to test the performance of our proposed method. Certainly, the point position is not estimated directly by our proposed method, but the purpose of the focal length and absolute orientation estimation in our method is 3D measurement, such as point position and 3D reconstruction. The absolute position of a point is generally measured by binocular vision, based on two cameras, after intrinsic and extrinsic camera parameter estimation, including the focal length and camera pose. When the intrinsic and extrinsic camera parameters are known, the least square method can be used to estimate the point position, and then the relative position error can be given, which is very simple. We can see that the key step of the point position estimation is the intrinsic and extrinsic camera parameter estimation, i.e., the focal length and absolute orientation in this paper. Therefore, the accuracy of the absolute orientation and focal length estimation directly affects the relative position error of points, and in turn, the relative position error can reflect the accuracy of the absolute orientation and focal length estimation with our proposed method. Moreover, the relative position error can be measured in our lab, since the ground truth of a point position can be given by the total station, and the measured value can be given using binocular vision with our proposed method. 

In addition, the ground truth of a point position is known, and then we can obtain the reprojection, based on the standard pinhole camera model [18], with the focal length and absolute orientation measured by our proposed method. The reprojection is the measured value of the imaging position and the ground truth can be obtained by corner detection from the real images. Therefore, the reprojection error is affected by the focal length and absolute orientation estimation, and in turn, the reprojection error can reflect the accuracy of the focal length and absolute orientation estimation with our proposed method.

Therefore, indirect analysis and comparison, for testing the performance of our method with real images, are practicable. Moreover, in this paper we use relative position and reprojection error to reflect the error of the focal length and absolute orientation estimation when the focal length and absolute orientation cannot be directly and accurately measured using direct physical measurement in the lab. The experiments and results with real images are as follows.

**Figure 11:**
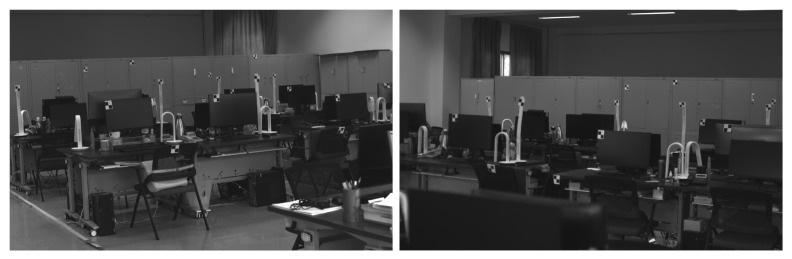
Real images form two cameras. Some control points were placed and measured using a total station.

In this section, real images were captured using two cameras, and then we tested our proposed method with them. Some control points were placed in these two camera fields of view, as shown in Figure 11.

These control points and the camera positions were measured as the ground truth using a total station (NTS-330R, measuring precision better than 0.5 cm). Since we did not know the ground truth of the camera pose in the real scenarios, the accuracy of the focal length and absolute orientation was not compared directly. In this paper, the accuracy of the absolute pose and focal length estimation is, thus, demonstrated by measuring the relative position and reprojection error of these known control points.

Then two 2D–3D point correspondences for our proposed method, three 2D–3D point correspondences for Kneip’s method, and four 2D–3D point correspondences for the GP4Pf were selected from these known control points to estimate the camera pose and focal length. Finally, we measured the relative position and reprojection of the rest of the control points using binocular vision and reported the average relative positional error between the ground truth and the measured values; the average reprojection error between the position in the real image and the measured value in Table 2.

From Table 2, according to the relative position error and reprojection error, we can observe that our proposed method performed better than Kneip’s method and GP4Pf, which shows our proposed method can work well in real scenarios.

At the beginning of Section 2, we assumed that the skew was zero, the aspect ratio of the pixels was one, and the principal point was the center of the image for our proposed method. Since we do not know the ground truth of the skew and the aspect ratio in real scenarios, the error of these assumptions cannot be directly discussed. However, the relative position and reprojection error in real images can indirectly show that our method can obtain good results under these assumptions. Actually, the relative position error directly reflects the total error introduced by our algorithm model and these assumptions. The relative position error was 0.39%, which is low and can meet the actual application requirements. We can see the relative positional error includes the error of these assumptions and, therefore, the error of these assumptions was less than 0.39%, which shows that these assumptions can yield good results in a real scenario experiment, even though they are not strictly true.

## 4. Discussion

Orientation and focal length estimation is one of the key steps in computer vision, photogrammetry, SLAM, and SfM. In this paper we propose an efficient closed form solution to the absolute orientation problem with unknown focal length and two 2D–3D point correspondences. The problem can be decomposed into two sub-problems and can be solved with angle constraints. A quadratic equation of one variable is solved to determine the focal length, and then a geometric approach is used to determine the absolute orientation, which is different from the existing orientation estimation solvers.

### 4.1. Differences and Advantages

In this paper, our core contribution is to use fewer 3D control points, for both absolute orientation and focal length estimation. With the development of measurement technology and the reduction in cost, more and more devices are being used to obtain partial pose parameters as prior knowledge, which is the reason why we performed our work with a known camera position. Our proposed method only needs two 3D control points and can estimate both pose and focal length. In contrast, the existing P3P solvers need three 3D points and can only estimate camera pose. 

Our proposed method uses partial pose parameters and, hence, can use fewer 3D control points. These partial pose parameters, i.e., camera position, are measured with high precision using RTK or total station (e.g., NTS-330R in Section 3), which is a reason why our proposed method performs better in terms of numerical stability and noise sensitivity.

The P3P solvers in previous studies used an iterative algorithm or needed to solve systems of quadratic or quartic polynomial equations; however, our proposed method only uses a geometric approach with angle constraints. This is another reason why our proposed method performs better in terms of numerical stability, noise sensitivity, and computational speed. In addition, the existing P3P solvers all have up to four possible solutions and need an extra point to give a single solution, which is also a main reason why our proposed method has a faster computational speed.

Our proposed method uses the camera position as the prior knowledge, which is different from the existing methods. Therefore, the camera position is important and we have analyzed the effect of error in the camera position on the estimation of the absolute orientation and of the focal length, as shown in Section 3.1.1. In geometric derivation, the camera position error contributes error to the angle in Equation (6) when we estimate the focal length. However, the camera position error is low, because of high-accuracy measurement using RTK or total station, which means that the error of angle in Equation (6) is very low. This is the reason why our proposed method still yields good results even though camera positional error exists.

As shown in Section 3, because of the lower noise sensitivity in rotation and translation error, our proposed method gives better result in terms of the reprojection error. It should be noted that the Harris algorithm [49] was used for feature point extraction in real images, and its precision is below 0.2 pixels. Hence the reprojection error in real images matches that in the synthetic data of a 0.2 pixel noise. In addition, an ideal focal length was used for the synthetic data and a focal length directly written on the lens, which has a small error, was used for real images. This is a reason why the reprojection error with synthetic data was slightly smaller than that in the real images. Finally, the higher precision in focal length and absolute orientation estimation led our proposed method to have results, in terms of the relative position error in binocular vision.

In brief, our proposed method has the following advantages: (1) Only two 3D points are needed to estimate the absolute orientation and focal length; (2) It gives a single solution and has no multi-solution phenomenon; (3) It performs better, in terms of numerical stability, noise sensitivity, computational speed, and robustness to camera position noise; and (4) It obtains better results, both with synthetic data and real images.

### 4.2. Future Work

Our proposed method has to use a positioning device (e.g., RTK, total station) to obtain the camera position and, as described in Section 1, some existing methods use the known vertical direction to obtain some orientation information using IMUs. Those methods can all use fewer 3D points to estimate camera pose than the existing P3P solvers. This may inspire us to use both camera position and vertical direction for pose and partial intrinsic parameter estimation in the future. This idea may lead to a faster and more efficient method.

Another work that will be completed in the future is to use a camera with a positioning device in practice, such as SfM and 3D reconstruction with the RANSAC algorithm [50]. The superior computational efficiency of our proposed method is particularly suitable as a RANSAC outlier rejection step.

## 5. Conclusions

We have proposed an efficient closed-form solution to the absolute orientation problem for a camera with unknown focal length from two 2D–3D point correspondences and the camera position. In the original state, the camera frame and the two 2D image points are known, and the world frame and the two 3D control points are also known. However, the 2D–3D point correspondences are unknown in the original state. Our main process is to rotate the original camera frame and world frame to make the camera position, 2D image point, and 3D control point collinear, and then obtain two 2D–3D point correspondences geometrically in the final state. Finally, the absolute orientation can be estimated based on the known camera frame, the known world frame in the original state, and the rotation angles. Before this, the focal length is estimated using angle constraint. 

By decomposing the problem into two sub-problems and solving them with angle constraints, only two 2D–3D point correspondences are needed to estimate the focal length and absolute orientation, and a single solution can be given with our method. The geometric derivations are easy to understand and significantly improve the performance. Experimental results show that our proposed method works well with synthetic data and real scenarios. It is particularly suitable for estimating the focal length and orientation of a zooming digital camera with fixed position or with a positioning device mounted on it.

## Figures and Tables

**Table 1:** Computational time.

Method	Proposed Method	Kneip’s Method	GP4Pf
Computational time	0.543 ms	0.556 ms	2.683 ms

**Table 2:** Relative position error and reprojection error for real images.

Method	Proposed Method	Kneip’s Method	GP4Pf
Relative position error/%	0.39	0.47	1.37
Reprojection error/pixel	0.36	0.56	0.78

## Data Availability

The data presented in this study are available in the manuscript.
